# PCK1 as a target for cancer therapy: from metabolic reprogramming to immune microenvironment remodeling

**DOI:** 10.1038/s41420-024-02240-8

**Published:** 2024-11-22

**Authors:** Na Liu, Xiao-ren Zhu, Chang-ying Wu, Yuan-yuan Liu, Min-bin Chen, Jin-hua Gu

**Affiliations:** 1 Department of Radiotherapy and Oncology, Affiliated Kunshan Hospital of Jiangsu University, Kunshan, China; 2 Department of Intensive Care Medicine, Chongqing People’s Hospital, Chongqing, China; 3 Clinical Research and Lab Center, Affiliated Kunshan Hospital of Jiangsu University, Kunshan, China; 4 Department of Clinical Laboratory, Kunshan First People’s Hospital, Affiliated to Jiangsu University Kunshan, Kunshan, China

**Keywords:** Developmental biology, Prognostic markers

## Abstract

Recently, changes in metabolites and metabolism-related enzymes related to tumor cell proliferation, metastasis, drug resistance, and immunosuppression have become a research hotspot, and researchers have attempted to determine the clinical correlation between specific molecular lesions and metabolic phenotypes. Convincing evidence shows that metabolic reprogramming is closely related to the proliferation, invasion, metastasis, and poor prognosis of malignant tumors. Therefore, targeting metabolic reprogramming is a new direction for cancer treatment. However, how molecular alterations in tumors contribute to metabolic diversity and unique targeting dependencies remains unclear. A full understanding of the underlying mechanisms of metabolic reprogramming in cancer may lead to better identification of therapeutic targets and the development of therapeutic strategies. Evidence for the importance of PCK1, a phosphoenolpyruvate carboxykinase 1, in tumorigenesis and development is accumulating. PCK1 can regulate cell proliferation and metastasis by remodeling cell metabolism. Additionally, PCK1 has “nonclassical” nonmetabolic functions, involving the regulation of gene expression, angiogenesis, epigenetic modification, and other processes, and has an impact on cell survival, apoptosis, and other biological activities, as well as the remodeling of the tumor immune microenvironment. Herein, we provide a comprehensive overview of the functions of PCK1 under physiological and pathological conditions and suggest that PCK1 is a potential target for cancer therapy. We also propose a future exploration direction for targeting PCK1 for cancer therapy from a clinical perspective. Finally, in view of the collective data, the results of our discussion suggest the potential clinical application of targeted PCK1 therapy in combination with chemotherapy and immunotherapy for cancer treatment.

## Facts

- PCK1 is widely involved in the reprogramming of glucose and lipid metabolism in cancer.
- PCK1 plays non-metabolic functions, such as angiogenesis, epigenetic modification, and immune microenvironment remodeling.
- The expression of PCK1 in cancer is tissue-specific.
- Targeting PCK1 in combination with chemoimmunotherapy may has excellent potential for clinical application in cancer therapy.

## Open questions

- Is there crosstalk between PCK1-mediated metabolic and nonmetabolic regulation in cancer development and progression?
- Whether PCK1 is binary in the remodeling of the immune microenvironment?
- Whether the combination of PCK1 with antiangiogenic therapy in cancer improves efficacy?

## Introduction

Since Hanahan and Weinberg published “The Hallmarks of Cancer” in 2000, researchers have been studying cancer increasingly deeply [1]. Currently, the number of cancer hallmarks has increased from six to fourteen, and one of these hallmarks is cell metabolic reprogramming [2]. For cancer cells to reproduce, they need a constant stream of rapidly synthesized large molecules [3]. Consequently, cancer cells often demonstrate a heightened rate of aerobic glycolysis, which is commonly referred to as the Warburg effect [4, 5]. Glucose, amino acid, and lipid metabolism play important roles in tumor progression, and the metabolism of these substances is regulated by metabolic enzymes and related signaling pathways [5–7]. Therefore, related metabolic enzymes are often dysregulated in cancer and have gradually become targets for cancer treatment [5–7]. Currently, several studies have been conducted to evaluate the therapeutic potential of targeting different metabolic enzymes involved in tumor metabolism [8–11]. The targeting of nucleotide metabolism by drugs has become integral to the treatment of cancer [8, 9]. Additionally, inhibitors of several key metabolic enzymes, such as IDH1(Isocitrate Dehydrogenase 1), IDO1((Indoleamine 2,3-dioxygenase 1), GPX4(Glutathione Peroxidase 4), and NAMPT(Nicotinamide phosphoribosyltransferase), have shown good anti-tumor effects [10–13]. The revolution of targeted metabolic therapy for cancer is about to begin.

The cell’s main energy source is glucose, and under aerobic conditions, the cell degrades glucose molecules into water and carbon dioxide through a series of reactions, and the energy stored in the molecules is “realized” into adenosine triphosphate (ATP) [14]. ATP is the energy currency needed for life to grow and reproduce [15–17]. Glucose degradation occurs in two stages: glycolysis in the cytoplasm, where glucose is converted to pyruvate; the tricarboxylic acid (TCA) cycle and oxidative phosphorylation in the mitochondria [17, 18]. Gluconeogenesis is the reverse process of glycolysis [19]. Phosphoenolpyruvate carboxykinase 1 (PCK1) is the first rate-limiting enzyme in the gluconeogenic pathway and catalyzes the conversion of oxaloacetate (OAA) to phosphoenolpyruvate (PEP) [19]. There is growing evidence that PCK1 mediates cancer development through metabolic and nonmetabolic processes in specific cases [19–22]. In this study, the complex associations between PCK1 and metabolic and nonmetabolic processes in tumors are reviewed, and the influence of changes in PCK1 on the metabolic diversity and targeted dependence of the whole tumor is emphasized, which provides a new perspective for improving cancer diagnosis and treatment.

## Overview of the biological characteristics, distribution, expression, and function of PCK1

In mammals, there are two PEPCK isoenzymes: the cytoplasmic subtype PCK1 and the mitochondrial subtype PCK2 (Phosphoenolpyruvate carboxykinase 2) [23]. Here, we mainly outline PCK1. currently, nearly 900 articles have been published on PCK1, with topics centered on metabolic disorders, inflammatory diseases and tumors [24–28]. Recently, in the context of the Warburg effect, PCK1 has shown its critical role in meeting the rapid growth, proliferation, and metabolic demands of tumor cells [29–32]. The study of PCK1 not only contributes to the understanding of the metabolic reprogramming mechanism of tumor cells, but also provides new potential targets for tumor diagnosis and treatment.

In terms of distribution, PCK1 is expressed in the liver, kidney, gastrointestinal tract, and adipose tissue [33]. Activation of the cAMP/AMPK signaling pathway under external conditions, such as hypoxia and starvation, significantly increased PCK1 mRNA expression [34]. Post-translational modifications (PTMs) of the PCK1 protein, such as acetylation, phosphorylation, sumoylation, and ubiquitination, affecting its protein stability have been continuously found in diseases, including cancer [35–40]. When PCK1 is stimulated by the complex and heterogeneous tumor microenvironment (TME), its expression is often dysregulated [41–43]. However, PCK1 expression is different in different tumors, possibly due to differences in organ tissues [41–43]. Functionally, PCK1 is the key rate-limiting enzyme that regulates gluconeogenesis, converting oxaloacetic acid to PEP, which is involved in a variety of anabolic pathways, including gluconeogenesis, glycerogenesis, serine synthesis and so on [44]. Additionally, PCK1 induces the entry of noncarbohydrate sources into glycolysis and acts as a key regulator of hypotrophic adaptation [44]. Surprisingly, in recent years, PCK1 has been shown to be involved not only in metabolic regulation but also in protein kinase, proangiogenic factor, immune response enhancer, epigenetic regulator, and other processes involved in various physiological and pathological processes, especially cancer [21, 22, 28, 45, 46]. In the following sections, we will specifically investigate the role and transformation of PCK1 in various cancers.

## PCK1 plays diverse roles in cancer development

**Fig. 1:**
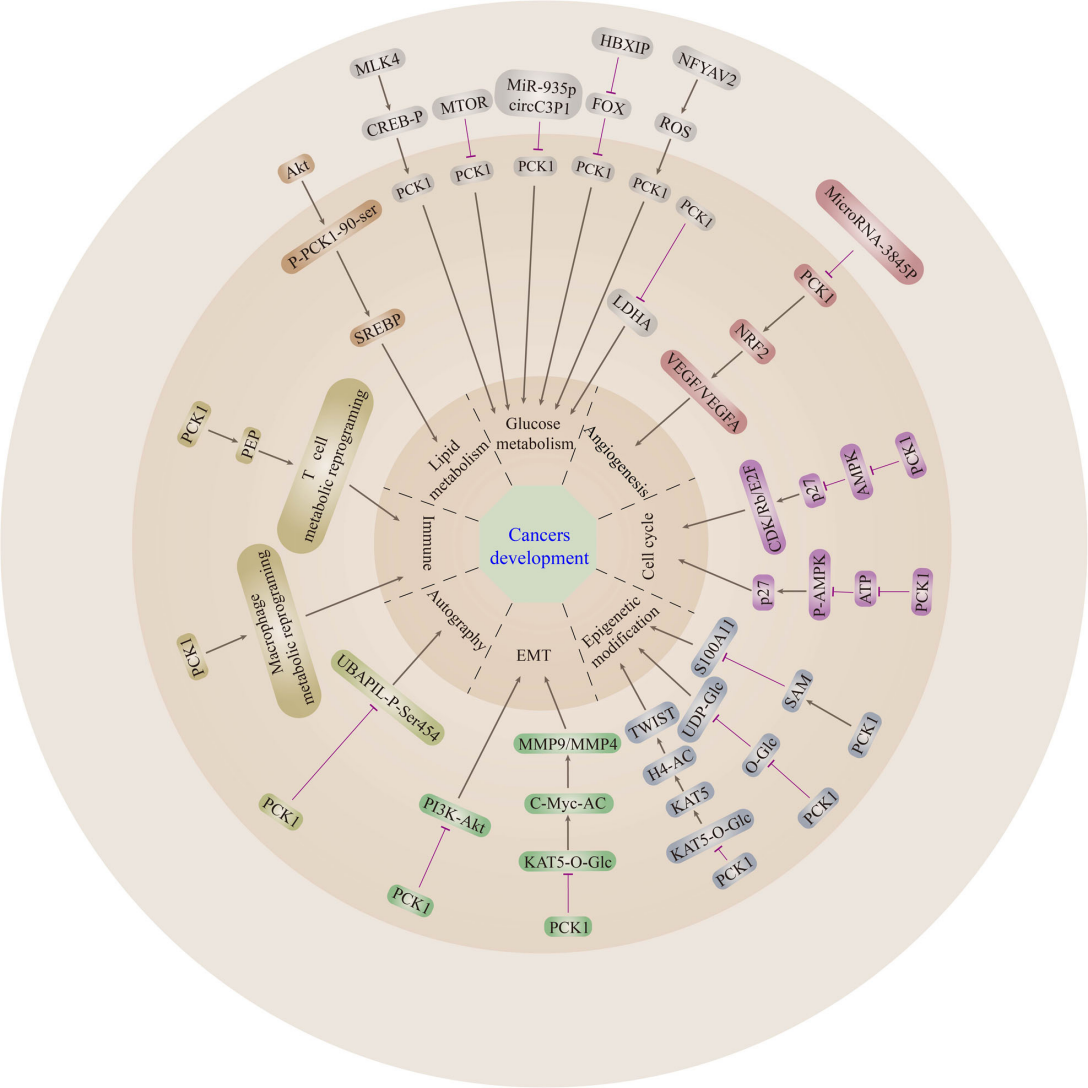
Schematic representation of the effect of PCK1-mediated signaling on cancer development. Please see the text for specific details. arrows indicate positive effects. perpendicular bars indicate negative effects. (O-Glc, O-GlcNAcylation; AC, Acetylate; P, phosphorylate; Ser, Serine).

PCK1 is widely involved not only in glycolipid metabolism but also in immune regulation, angiogenesis, epigenetic modification, and other processes (Fig. 1). However, the crosstalk effect among them is unknown. Consequently, elucidation of the tumor-specific regulation of PCK1 metabolism and nonmetabolism may help better identify therapeutic targets and develop therapeutic strategies. It is indisputable that a rational cancer treatment strategy should consider targeting multiple pathways simultaneously, with specific treatments targeting carcinogenic agents or related signaling pathways.

### PCK1 and glucose metabolism

**Fig. 2:**
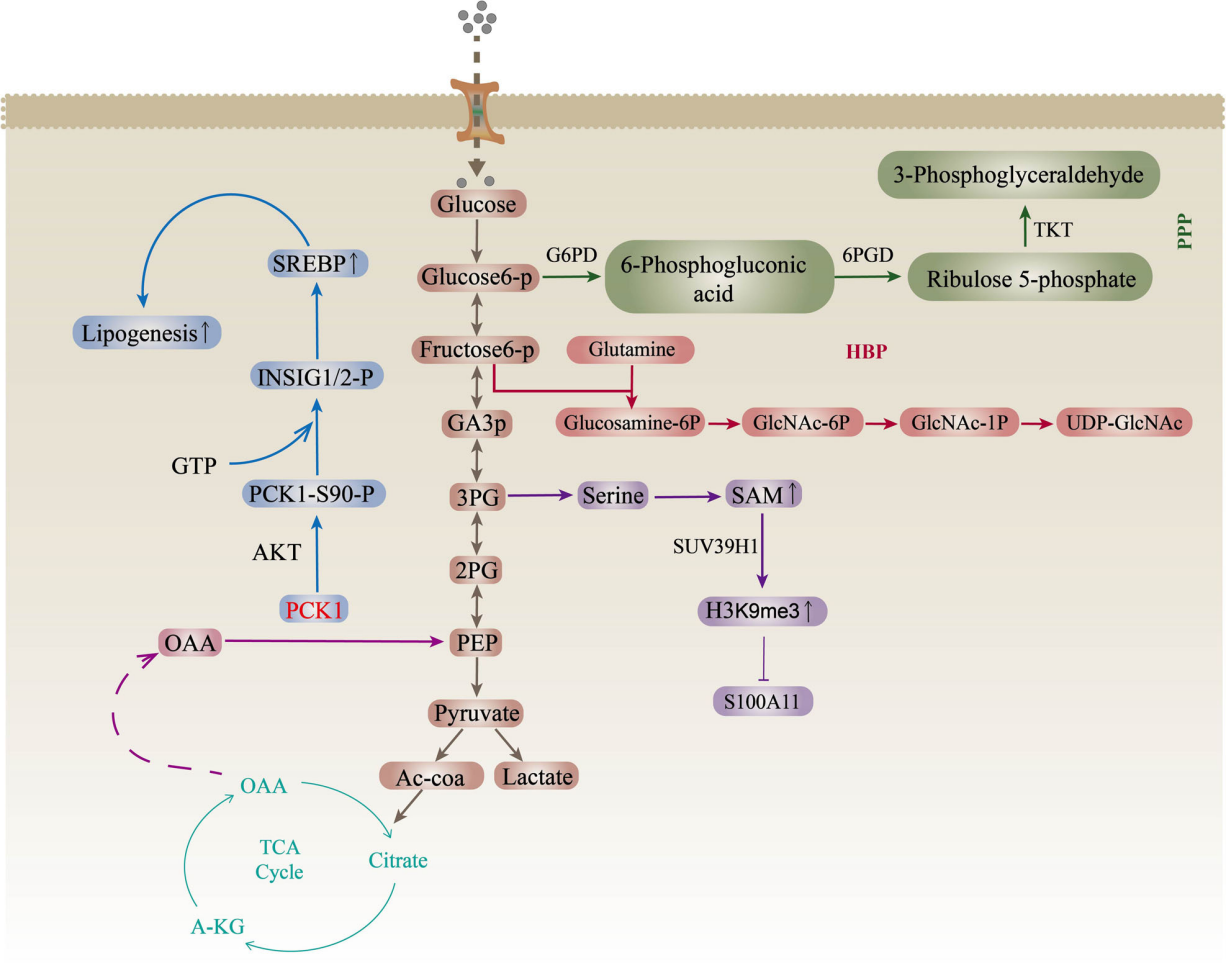
Schematic representation of PCK1 involved in glucose and lipid metabolism. Please see the text for specific details. arrows indicate positive effects. perpendicular bars indicate negative effects. (AC-coa, Acetyl-CoA; P; phosphate, S, Serine; GA3p, glyceraldehyde-3-phosphate; 3PG, 3-Phosphoglycerate; 2PG, 2-Phosphoglycerate；OAA, Oxaloacetete; PPP, Pentose phosphate pathway, PPP; HBP, Hexosamine biosynthesis pathway;G6PD,Glucose-6-phosphate dehydrogenase; 6PGD, 6-phosphogluconate dehydrogenase; TKT, Transketolase, UDP-GlcNAc, Uridine diphosphate-N-acetylglucosamine).

The gluconeogenic pathway, an inverse process of glycolysis, was previously generally thought to be inhibited in tumors to enhance glycolysis to provide energy for cancer [19]. However, recently, numerous studies have revealed that the key enzymes involved in gluconeogenesis also have powerful protumor or anti-tumor functions [19, 46, 47]. PCK1 catalyzes the conversion of oxaloacetic acid to PEP, and intracellular PEP is involved in various anabolic pathways, including gluconeogenesis, glycerogenesis, serine synthesis, and the pentose phosphate pathway [20, 44] (Fig. 2). Additionally, PEP maintains the metabolic flux of TCA cycle by removing excess oxaloacetic acid and replenishing the required TCA intermediates, regulating the TCA cycle and cell proliferation [48, 49]. Consequently, tumor cells with high PCK1 expression can use nonsugar energy, such as lactic acid and glutamine, to meet the needs of cancer cell proliferation in an environment of glucose deficiency [48]. Therefore, tumor cells with high PCK1 expression can use nonsugar energy sources such as lactic acid and glutamine to meet the needs of cancer cell proliferation in a glucose-deficient environment, and PCK1-activated gluconeogenesis provides glycolytic intermediates in cancer cells for growth and antioxidant defense [48, 49]. PCK1 coordinates the ability of cells to absorb and metabolize glucose (glycolysis) for energy and anabolic purposes [48, 49].

To meet the biological energy, biosynthesis, and redox requirements of malignant tumors, tumors change their metabolic pathway and increase their glycolytic capacity by 20–30 times [5, 49]. Increased glycolysis promotes the transfer of glycolytic intermediates into various biosynthetic pathways, including those that produce amino acids and nucleosides, and promotes the biosynthesis of macromolecules and organelles required for cancer cell proliferation [44]. Simultaneously, gluconeogenesis is inhibited [44]. PCK1 inhibits Warburg effect-related pathways in hepatorenal cell carcinoma by preventing glucose from entering the glycolytic pathway, thereby inhibiting tumor cell growth and promoting tumor cell death [41, 42]. Interestingly, increased expression of the PCK1, a key rate-limiting enzyme for gluconeogenesis, activates both gluconeogenesis and glycolysis in non-gluconeogenic tissue cancers [50, 51]. Notably, glucose concentrations in tumor tissues are typically 3–10 times lower than those in neighboring normal tissue. Consequently, cancer cells are in a state of starvation and require high levels of glycolysis and gluconeogenesis. At this time, tumor cells maintain their energy requirements through PCK1-mediated metabolic reprogramming in response to changes in the microenvironment and bioenergy fluctuations [52]. In general, PCK1 combines gluconeogenic-tricarboxylic acid-glycolysis to play a complex synergistic regulatory role in cancer.

### PCK1 and lipid metabolism

In the TME, the availability of nutrients is a constantly changing facto [44]. Disruption of fatty acid (FA) and cholesterol metabolism emerging as one of the most prominent metabolic alterations in cancer [44]. Cancer cells obtain the energy, biological membrane components, and signaling molecules required for cancer cell survival and response to the TME and cancer treatments through lipid metabolism [53, 54]. Lipogenesis is regulated by the SREBP(Sterol-regulatory element binding protein) family (SREBP1a, SREBP1c and SREBP2) [55]. In tumor cells activated by tyrosine kinase receptor or the KRAS oncogene, AKT(Protein Kinase B, PKB) phosphorylates serine at 90 of PCK1, and phosphorylated PCK1 translocates to the endoplasmic reticulum and loses its original gluconeogenic metabolic enzyme function [20, 28] (Fig. 2). Instead, PCK1 acquires its protein kinase function, and the phosphorylation of INSIG1/2(insulin induced gene 1/2) with GTP(Guanosine Triphosphate) as the phosphate group donor disrupts the interaction with SCAP(SREBP cleavage‐activating protein) [20, 28]. Free SCAP escorts SREBPs to the Golgi body, and the activation of SREBPs leads to the transcription of adipogenic genes to facilitate lipid synthesis, thus promoting cancer development [20, 28]. The role of PCK1 as a protein kinase regulating lipogenesis has been demonstrated in liver cancer, non-small cell lung cancer (NSCLC), and esophageal cancer [20, 38, 56]. Accordingly, targeting PCK1 to inhibit lipid production is a promising strategy for treating related cancers. Additionally, male mice with liver PCK1 deficiency showed liver lipid disorders and liver damage on a normal diet, while fibrosis and inflammation increased in mice fed drinking water containing fructose and glucose (HFCD-HF/G) on a high-fat diet [26]. Adeno-associated virus forced expression of hepatic PCK1 improves metabolic-associated fatty liver disease in male mice. Consequently, PCK1 deficiency stimulates lipid gene expression and lipid synthesis, leading to metabolic-associated fatty liver disease [26]. Studies on the nonclassical and nonenzymatic functions of PCK1 have improved our understanding of tumor metabolic enzymes.

### PCK1 assists in immunity

It is well known that dysregulated metabolism in the TME drives cancer cell growth, but it is undeniable that dysregulated metabolism also affects immune cell metabolism and function [57, 58]. Therefore, cancer cells and immune cells struggle in metabolism to survive, and the cells that metabolize the most glucose have a survival advantage [59]. It has been reported that cancer cells, as a whole, account for approximately two-thirds of glucose intake, myeloid cells account for one-third of glucose intake, and other immune cells account for a negligible proportion of glucose intake [59]. Low-sugar and high-lactic acid environments in tumors affect T-cell function [60–62]. Malignant results, such as the active proliferation of tumor cells and the progression of metastasis, are partly because tumor T cells are deprived of glucose, resulting in decreased function and inability to destroy cancer cells [63, 64]. Studies have demonstrated that metabolic reprogramming of tumor T cells allows them to kill tumor cells more efficiently. Consequently, metabolic reprogramming of tumor-specific T cells may be an adjunct form of immunotherapy [62–64].

In 2015, scientists at Yale University proposed a new way to metabolically “reprogram” immune cells to boost immune responses [30]. Specifically, glucose is a nutrient necessary for T cells to function, and increased glycolytic activity in most tumors helps them to better evade T-cell immune surveillance. Moreover, T cells under these conditions show signs of glucose deprivation, including impaired IFN-γ(Interferon-gamma) and CD40L production [30]. Mechanistically, glycolysis in tumors controls T-cell Ca2 + -NFAT signaling, and effectors act through the glycolytic metabolite PEP [30]. PEP is a key metabolite that controls T-cell function. By increasing the production of PEP through PCK1 overexpression, T cells are metabolically reprogrammed, T-cell function is restored, anti-tumor immune function is strengthened, and tumor growth is limited [30]. In 2021, Huang Bo’s research group found that CD8+ memory T cells utilize unique glucose metabolism modes to form memory and maintain long-term activity [21]. Memory T cells are very active in gluconeogenesis, with high expression of cytoplasmic PCK1, a key rate-limiting enzyme of gluconeogenesis, which catalyzes the generation of oxaloacetic acid to glucose 6-phosphate [21]. Due to the lack of glucose-6-phosphatase, memory T cells do not convert glucose 6-phosphate into glucose [21]. Instead, glycogen is synthesized, and after decomposition, glucose 6-phosphate is generated, while glucose 6-phosphate enters the pentose phosphate pathway to increase the concentration of the reducing agent glutathione, quickly clearing free radicals in the cell, thus reducing oxidative damage to mitochondria and prolonging the life of memory CD8 + T cells [21]. Furthermore, when PCK1-overexpressing tumor-specific T cells were injected back into tumor-bearing mice, a stronger anti-tumor immunotherapy effect was obtained than that achieved with control cells [21]. PCK1 overexpression not only enhances its anti-tumor function but also prolongs the lifespan of memory CD8 + T cells through metabolic reprogramming of T cells [21]. These studies reveal a potential new approach to cancer immunotherapy. However, loss of PCK1 in macrophages contributes to metabolic reprogramming and M1 polarization of macrophages and increases the expression of the proinflammatory cytokines(IL-6, IL-1β, TNF-αand so on), leading to anti-tumor effects [65]. Due to the high levels of cytokines, the TME is heterogeneous, and PCK1 regulates the function of immune cells differently. Whether PCK1 regulates broader and fundamental immunomodulatory functions must be further studyed.

### PCK1 regulates epigenetic modifications

Epigenetic modification, which mainly includes DNA methylation, histone modification, and noncoding RNA modification, is a way to regulate gene expression without changing the DNA sequence [66]. PTMs of histones, such as methylation and acetylation, are dynamic and reversible PTM that regulate gene transcriptional activation or repression and are believed to play indispensable roles in the occurrence and development of many cancers [67, 68]. Recent studies have shown that there is a wide range of mutual regulation between metabolic reprogramming and epigenetic modifications [66]. The process of epigenetic modification requires the participation of metabolites, and the characteristic metabolic changes of cancer will affect the activity of epigenetic modifying enzymes and cofactors or the abundance of substrates by changing the level of metabolites, thus participating in epigenetic regulation [66]. Furthermore, changes in the expression or activity of epigenetic modifying enzymes can also have widely direct or indirect effects on cell metabolism [66]. Early studies showed that PCK1 can promote H3 histone methylation and inhibit H4 acetylation [69, 70]. Recently, PCK1 was reported to affect hepatocellular carcinoma (HCC) progression by regulating histone epigenetic modifications, revealing the crosstalk between metabolic reprogramming and epigenetic regulation [22]. In HCC, PCK1 promotes the generation of S-adenosylmethionine (SAM) through the serine synthesis pathway, and the methyltransferase SUV39H1 catalyzes SAM as a methyl donor to support H3K9me3 modification, thereby repressing the oncogene S100A11 [22] (Fig. 2). Additionally, lysine acetyltransferase 5 (KAT5) was found to be highly modified by O-GlcNAcylation in PCK1-missing HCC cells [41]. Further studies showed that PCK1 depletion stabilized KAT5 by inhibiting KAT5 ubiquitination through increasing KAT5 O-GlcNAcylation, which epigenetically activated TWIST1 expression through histone H4 acetylation [41]. Moreover, c-Myc acetylation enhances the expressions of MMP9(matrix metalloproteinase 9) and MMP14(matrix metalloproteinase 14), thereby promoting epithelial-mesenchymal transition (EMT) in HCC [41]. PCK1 is a linker for the crosstalk of metabolic reprogramming, PTM, and epigenetic modification in HCC.

### Role of PCK1 in angiogenesis

The unlimited proliferation of cancer cells accelerates the consumption of nutrients and the insufficiency of the vascular system, resulting in a microenvironment of malnutrition, ischemia, and hypoxia [71, 72]. However, this situation is only temporary, and cancer cells activate various oncogenic signaling pathways to promote metabolism and angiogenesis to supply energy [72]. When oxygen and nutrient supplies are urgently needed, angiogenesis can be initiated under nutrient-poor conditions, specifically in ischemic and/or hypoxic environments [72]. The stimulation of a variety of growth factors, including vascular endothelial growth factor (VEGF) and many others, can also stimulate angiogenesis [72, 73]. Recent studies have disclosed that metabolism-related enzymes also play important roles in angiogenesis [74, 75]. Hepatocellular cell-derived extracellular vesicles encapsulated with microRNA-584-5p activate the NRF2(Nuclear Factor erythroid 2-Related Factor 2) signaling pathway by inhibiting the expression of PCK1 and increasing the expression of VEGF, VEGFA, VEGFR2, and other proangiogenic factors to promote angiogenesis in tumor tissues [74, 75]. Therefore, PCK1 plays a role in inhibiting cancer angiogenesis. However, PCK1 depletion effectively inhibits endothelial cell proliferation, migration, sprouting, and tube formation, while ectopic PCK1 overexpression accelerates endothelial cell proliferation, migration, sprouting, and angiogenesis [76]. PCK1 is a key molecule for normal vascular endothelial development, and loss of PCK1 disrupts endothelial function [76]. In vivo, endothelial knockout of PCK1 delayed retinal vasculature development in neonatal mice and impaired retinal angiogenesis in adult mice [76]. The roles of PCK1 in angiogenesis in the above two studies were completely opposite. The reason may be that one study was in cancers, and one study was in normal cellular blood vessels. PCK1, a metabolic enzyme, is largely affected by the internal and external environment.

## PCK1 and multiple malignancies

### Liver cancer

The liver is the most important metabolic organ in the human body [77]. Metabolic diseases such as nonalcoholic fatty liver disease (NAFLD), nonalcoholic steatohepatitis (NASH), and viral hepatitis have caused metabolic disorders and become the main risk factors for HCC development [78]. In recent years, research on metabolism as a target for treating liver cancer has been expanding [79, 80]. There is compelling evidence that tumorigenesis is driven by the reprogramming of cellular metabolism as a result of direct and indirect consequences of oncogenic mutations, particularly during HCC development [45, 81, 82]. The liver is the major site of gluconeogenesis, which is regarded as the reverse process of glycolysis and is widely believed to be inhibited in cancer [22, 41]. However, PCK1 gene overexpression antagonizes HCC by activating gluconeogenesis and inhibiting the glycolytic pathway [22, 41]. The mechanism underlying the dysregulated expression of PCK1 in HCC and its role as a tumor suppressor gene affecting the expression of related oncogenes or oncogenic signaling pathways have been explored. Before the development of HCC, P53, SHP-1(Src homology 2 domain-containing protein tyrosine phosphatase 1), ZBTB22(Zinc finger and BTB domain containing 22), and NFY(Nuclear Factor Y) can act as transcriptional coactivators of PCK1, controlling hepatic glucose production and regulating glucose homeostasis [83–86]. However, in HCC, PCK1 is dysregulated by multiple oncogenes, oncogenic signaling regulation, and tumor metabolism [22, 41, 45]. Numerous studies have confirmed that PCK1 is significantly downregulated in HCC and that PCK1 depletion inhibits apoptosis or induces cancer cell proliferation and metastasis [22, 41, 45]. PCK1 overexpression attenuated HCC cell invasion and metastasis [41]. HCC tissue or cell-derived miR-93-5P and circC3P1 inhibited PCK1 expression to inhibit gluconeogenesis in HCC while promoting glycolysis and the malignant progression of HCC [87, 88]. Additionally, HBXIP(Hepatitis B X-interacting protein) downregulates the transcription factor FOX (Forkhead-box) and inhibits PCK1 expression to inhibit gluconeogenesis and promote hepatocarcinogenesis [89]. However, in glucose-starved HCC cells, NFYAv2 is upregulated in response to glucose deprivation to induce high ROS levels and energy crises and to promote PCK1 transcriptional activity, ultimately leading to HCC cell death [31]. Consistent with this, forced expression of PCK1 in glucose-starved HCC cells induces sudden TCA cycle death, leading to energy crisis and oxidative stress [48]. Interestingly, a recent study showed that intermittent fasting regimens (fasting for 24 h two days a week) prevent and improve NASH and fibrosis, and limit the development of HCC; Specifically, PPARα and PCK1 are liver performers that produce beneficial effects in NASH during fasting; Overexpression of PCK1 reduces lipid accumulation and liver steatosis, preventing NASH transformation into liver cancer [27]. As a non-drug regimen for the prevention of liver cancer, intermittent fasting has important research value and application prospect [27]. This study not only deepens our understanding of the mechanism of liver cancer, but also provides new and targeted targets for future drug development [27].

The NRF2 signaling pathway is frequently activated in tumors and is involved in tumor proliferation, invasion, and angiogenesis [75]. Studies have revealed that hepatocellular cell-derived extracellular vesicles coated with microRNA-584-5p activate the NRF2(Nuclear Factor erythroid 2-Related Factor 2) signaling pathway by inhibiting the expression of PCK1 and increasing the expression of VEGF, VEGFA, VEGFR2, and other proangiogenic factors to promote angiogenesis in tumor tissues [75]. Additionally, knockdown or deletion of PCK1 activated the NRF2/Keap1 pathway to promote HCC cell proliferation [74]. PCK1 deficiency in HCC inactivates AMPK(Adenosine 5‘-monophosphate (AMP)-activated protein kinase), inhibits p27kip1 expression, and stimulates the CDK/Rb/E2F pathway to promote cell proliferation, thereby accelerating the cell cycle transition from G1 to S phase under glucose starvation conditions [90]. However, PCK1 overexpression reduced cellular ATP levels and enhanced AMPK phosphorylation and p27kip1 expression but decreased Rb phosphorylation, leading to cell cycle arrest at G1 [90]. Consequently, PCK1 expression can regulate the cell cycle and affect cancer progression.

Recently, studies have disclosed that PCK1 is involved in regulating epigenetic modifications to affect the progression of liver cancer [22]. The gluconeogenic enzyme PCK1 promotes the production of SAM through the serine synthesis pathway [22]. The methyltransferase SUV39H1 catalyzes SAM as a methyl donor to support H3K9me3 modification, thereby repressing the oncogene S100A11 [22]. Consequently, PCK1 deficiency or decreased expression in HCC induces S100A11 oncogene activation and promotes HCC progression [22]. Additionally, there is growing evidence that increased O-GlcNAcylation promotes tumorigenesis and metastasis, and high levels of O-GlcNAcylation have been detected in malignancies such as breast cancer and liver cancer [91, 92]. Importantly, loss of PCK1 may contribute to elevated o-glcn acylation levels in HCC [45]. Mechanistically, metabolic reprogramming of PCK1-miss HCC cells leads to oxaloacetate accumulation and increased synthesis of neuridine triphosphate, which promotes the biosynthesis of uridine diphosphate-N-acetylglucosamine (UDP-GlcNAc) [45]. Moreover, loss of PCK1 leads to inactivation of the AMPK-GFAT1 axis, which promotes UDP-GlcNAc synthesis and leads to increased O-GlcNAcylation levels [45]. KAT5 was found to be highly modified by O-GlcNAcylation in PCK1 knockout HCC cells [41]. PCK1 depletion stabilizes KAT5 by inhibiting KAT5 ubiquitination by increasing the O-GlcNAcylation of KAT5 [41]. Additionally, KAT5 O-GlcNAcylation epigenetically activates TWIST1 expression through histone H4 acetylation and enhances MMP9(Matrix metalloproteinase-9) and MMP14(Matrix metalloproteinase-9) expression through c-Myc acetylation, thereby promoting EMT in HCC [41]. These findings link the crosstalk between metabolic enzymes, PTMs, and epigenetic regulation in HCC, providing a rationale for multitarget cancer therapy. Xu et al. demonstrated that in tumor cells activated by the tyrosine kinase receptor or KRAS oncogene, AKT phosphorylates PCK1 at serine 90, leading to the ER translocation of PCK1 and its protein kinase function [28]. PCK1 phosphorylates INSIG1/2 using GTP as a phosphate donor to block its binding to intracellular lipids, thereby promoting the activation of the SREBP signaling pathway and lipid synthesis in tumor cells [28]. Overall, PCK1 has complex and diverse functions in HCC. As a key metabolic enzyme in gluconeogenesis and regulator of epigenetic modifications, PCK1 is known as a tumor suppressor gene. Hepatic cells largely express PCK1, which produces glucose through the gluconeogenesis pathway. However, malignant hepatocytes inhibit gluconeogenesis by downregulating PCK1 in favor of tumor glycolysis. When acting as a protein kinase, PCK1 is regarded as an oncogene. Therefore, whether PCK1 acts as a rate-limiting enzyme or a protein kinase in HCC needs to be clarified before it can be targeted for cancer therapy. Additionally, PCK1 can regulate its downstream target genes to affect angiogenesis in HCC. We propose that targeting PCK1 can be combined with chemoradiotherapy to increase the sensitivity of cancer cells to chemoradiotherapy by blocking the blood supply of cancer cells.

### Renal carcinoma

The kidney is one of the main sites of gluconeogenesis and participates in the regulation of glucose homeostasis in the body [93]. As in HCC, the downregulation of PCK1 expression in clear cell renal cell carcinoma (CCRCC) inhibited gluconeogenesis and increased glycolysis compared with adjacent paracancerous tissues [94, 95]. Clinically, low PCK1 expression is associated with poor prognosis in patients with CCRCC [42]. Clearly, PCK1 functions as a tumor suppressor in CCRCC. PCK1 inhibited CCRCC proliferation and tumor growth [42]. Mechanistically, PCK1 destabilizes lactate dehydrogenase A (LDHA) through post-translational regulation, suppressing the Warburg effect and tumor progression [42]. Interestingly, mTORC2 regulates PCK1 expression to control gluconeogenesis [95]. When MTOR is inhibited in HCC and RCC cells, glycolysis shifts to the gluconeogenesis pathway, PCK1 expression increases, gluconeogenesis increases, and cell proliferation decreases [95]. Simultaneous knockdown of the PCK1 and mTOR pathways can overcome the inhibition of cancer cell proliferation [95]. At present, studies on PCK1 in renal cancer are scarce, but the impact is large, suggesting that glucose metabolic reprogramming may be an effective target for renal cancer treatment. However, the mechanism of PCK1 in RCC needs to be further explored.

### colorectal cancer

PCK1 has been reported to be frequently upregulated in patients with metabolic syndrome and diabetes [25, 39]. Epidemiologic data suggest that in patients with CRC, diabetic patients treated with metformin show better clinical outcomes compared with those untreated with metformin [96, 97]. Additionally, metformin improved overall survival after surgery in CRC patients with diabetes [96, 97]. In KRAS-mutated CRC, upregulation of PCK1 transcript levels was detected, and high expression of PCK1 was associated with poor survival in CRC patients [50, 98]. Compelling evidence suggests that PCK1 is strongly expressed in CRC cells and that PCK1 promotes cancer cell proliferation by increasing the anabolic utilization of glucose and glutamine. Moreover, PCK1 knockdown has been shown to reduce glutamine utilization and the TCA cycle and inhibit cancer progression [50, 98]. Therefore, targeting the ability of CRC cells to utilize glucose and glutamine would provide significant therapeutic advantages. Importantly, the effects of PEPCK on glucose metabolism and cell proliferation are mediated in part by activating mTORC1 [99]. Additionally, PCK1 can drive CRC cell proliferation and liver metastasis colonization by enhancing nucleotide synthesis in a hypoxic environment [100]. However, recent studies have revealed that the expression level of PCK1 in CRC is significantly lower than that in control tissues, and its expression level is negatively correlated with tumor progression, while its overexpression inhibits CRC cell growth, and silencing PCK1 promotes tumor proliferation [43]. Mechanistically, PCK1 activates oncogene autophagy by downregulating the phosphorylation of ubiquitin-associated protein 2-like (UBAP2L) at Serine 454. Hence, CRC growth was inhibited [43].

Therefore, the expression level of PCK1 in CRC is controversial. Some studies have disclosed that PCK1 is expressed at low levels in CRC tissues compared with adjacent tissues, and some studies have shown that PCK1 is highly expressed [43, 50, 98]. More surprisingly, PCK1 plays a role in promoting cancer proliferation and metastasis and plays an inhibitory role in the proliferation and metastasis of CRC. What accounts for these diametrically opposed results? Through in-depth analysis and interpretation of these studies, the following conclusions can be drawn: (1) PCK1 was previously reported to be upregulated in KRAS-mutated CRC, and PCK1 is highly expressed in CRCs with P53 mutations. In CRC without P53 mutation, PCK1 is inhibited by P53, and its expression is significantly decreased. Therefore, when using PCK1 as a therapeutic target in CRC research, it is crucial to detect whether the gene is mutated [101]. (2) The metabolic abnormalities caused by different stages of CRC and different states of cancer cells affect PCK1 expression and glucose metabolism reprogramming. (3) There may be translocation of PCK1 in cells, resulting in differences in the expression and function of PCK1. Of course, the above is only a conjecture, and further experiments are needed to determine the specific reason.

### Lung cancer

In the past decade, studies have continuously revealed the impact of metabolic reprogramming pathways on the progression of lung cancer, suggesting that metabolic reprogramming pathways are important potential targets for new targeted therapies for lung cancer [102, 103]. In NSCLC, studies have shown that glycolysis and gluconeogenesis are activated and is correlated with prognosis [51]. Gluconeogenesis provides intermediates for glycolysis in cancer cells and is often activated along with glycolysis when glucose availability is limited or proximal glycolysis is inhibited [51]. In the future, the co-activation of gluconeogenesis and glycolysis should be considered in the therapeutic strategies targeting cancer metabolism to address the ineffectiveness of glycolytic inhibitors alone. Clinically, high PCK1 expression has been reported to predict poor survival in patients with lung adenocarcinoma [104]. Mechanistically, MLK4(Mixed lineage kinase 4) regulates PCK1 expression at the transcriptional level by phosphorylating the transcription factor CREB to maintain aerobic glycolysis and promote cancer cell survival [104]. Hence, blocking the MLK4-PCK1 axis may affect glucose metabolism. Surprisingly, PCK1 promotes lipid synthesis through the same mechanism as HCC in lung cancer. Akt-dependent and PCK1-mediated phosphorylation of INSIG1/2 and nuclear SREBP1 expression are associated with NSCLC progression [38]. PCK1 acts as a protein kinase to activate SREBP1 to increase lipid synthesis and promote tumor growth in NSCLC [38].

### Melanoma

PCK1 is expressed in human primary melanoma tumor-repopulating cells (TRCs) [105]. However, PCK1 does not play a role in enhancing gluconeogenesis in B16 TRCs [105]. In contrast, PCK1 promotes the flow of carbon from glucose to lactate from TRCs to promote glucose consumption [105]. Further experiments confirmed that PCK1 has a cancer-promoting effect [105]. Knocking down PCK1 or reducing its enzymatic activity slowed TRC growth and hindered tumorigenesis [105]. In summary, the differential expression of the gluconeogenic enzyme cytoplasmic phosphoenolpyruvate carboxykinase in B16 TRCs not only promotes glucose collateral metabolism but also enhances TRC glycolysis, hence endogenesis TRCSs with the ability to repopulate tumors [105]. Upregulation of PCK1 expression is a key metabolic feature of tumorigenic TRCS, thus providing a potential target for melanoma therapy [105]. Additionally, PCK1 is involved in developing melanoma drug resistance [106]. High PCK1 protein expression was detected in vilofinib-resistant melanoma cells, and further studies confirmed that PCK1 overexpression promoted cell proliferation and invasion, and reduced verofinib-induced oxidative damage [106]. The role of PCK1 in non-gluconeogenesis in melanoma provides new hope for its treatment and has become a key metabolic target for melanoma treatment.

### Other tumors

In addition to the occurrence and development of the above cancers, prognosis is closely linked to PCK1, and the development and prognosis of pancreatic cancer, esophageal cancer, gastric cancer, prostate cancer, and other cancers are also related to PCK1 [56, 107]. The research of our group shows that PCK1 is overexpressed in pancreatic cancer and that PCK1 silencing significantly inhibits cancer cell proliferation, migration, and invasion and induces cell apoptosis [107]. Further research found that ectopic overexpression of PCK1 mediated Akt activation to promote pancreatic cancer proliferation and metastasis [107]. Additionally, as an AKT-dependent protein kinase, PCK1 promotes SREBP1-dependent lipogenesis, which is positively correlated with esophageal cancer progression and metastasis [56]. as report goes that the expression levels of AKT pS473, AKT-regulated PCK1 pS90, and nuclear SREBP1 were higher in esophageal squamous cell carcinoma specimens than in adjacent nontumor tissues [56]. AKT activation depends on the activation of SREBP1 by the PCK1 protein kinase to mediate the tumor lymph node and metastasis of esophageal squamous cell carcinoma [56]. In gastric cancer, increased PCK1 levels promote the TCA cycle, activate the RAS/extracellular signal-regulated kinase (ERK) signaling pathway, induce MMP-9 expression, and eventually lead to gastric cancer metastasis [108]. Patients with castration-resistant prostate cancer (CRPC) often exhibit neuroendocrine differentiation, high mortality [109]. Studies have displayed that PCK1 upregulation supports CRPC cell proliferation and promotes the expression of neuroendocrine markers that favor CRPC development. Therefore, targeting PCK1 reduces the neuroendocrine phenotype of prostate cancer cells and inhibits their growth [32]. Importantly, two clinically used compounds (nilotinib and lapatinib) target PCK1 and cause AR-negative and NE-like PCa(prostate cancer) death [32]. Consequently, therapeutic strategy targeting PCK1 may be effective against PCa by modulating PCK1-driven metabolic responses.

## The prospect of targeting PCK1 for cancer therapy

Earlier studies revealed that 3-mercaptocarbamate (MPA) is a potent glucose-lowering agent that inhibits glucose synthesis by specifically inhibiting PEPCK in the gluconeogenic pathway [110]. 3-MPA, a PCK1 inhibitor, has been shown to significantly inhibit tumor growth and metastasis in mice with melanoma, CRC, or breast cancer [52, 100, 106]. Using MPA as the initial chemical structure, a more selective inhibitor, 3-[(carboxymethyl) thio-pyridinate] acid (CMP), has been prepared [111]. The use of PCK1 inhibitors to improve glucose homeostasis in anticancer therapy underscores the potential importance of this pathway as a novel therapeutic target. Interestingly, studies have used clinical compounds (nilotinib and lapatinib) that also target PCK1 to inhibit tumor growth in AR-negative and NE-like PCa cell models [32]. Currently, some clinically available drugs that target PCK1 to modulate PCK1-driven metabolic responses are effective against cancer progression. Examples include metformin and dexamethasone [90, 112, 113]. Metformin, an AMPK activator, compensates for AMPK inactivation caused by PCK1 loss by activating AMPK to induce cell cycle arrest and inhibit tumor growth [90, 112]. To date, clinical trials of metformin with or without other drugs as a treatment for cancer patients are ongoing [114]. Similarly, dexamethasone restored gluconeogenesis and induced PCK1 expression in malignant cells, which inhibited HCC growth in mice [113]. These findings shed light on PCK1 and its regulatory metabolic pathways as potential targets in cancer therapy. In particular, it should be noted that in view of the inconsistent effects of PCK1 in gluconeogenic and non-gluconeogenic tumors, different therapeutic strategies should be considered in future targeted tumor therapy for different tumor types. For example, PCK1 inhibitors should be used in tumors of non-gluconeogenic organs (colorectal cancer, lung cancer, melanoma, breast cancer, pancreatic cancer, stomach cancer) to exert anticancer effects, while PCK1 expression should be promoted in tumors of gluconeogenic organs (HCC and RCC) to inhibit tumor progression.

Multitarget combination therapy is the first choice for cancer treatment. Studies have demonstrated that targeting PCK1 in combination with chemotherapy can not only solve the problem of drug resistance but also play a synergistic role in increasing the anticancer effect. For example, in HCC, the use of auranofin, a TXNRD1(Thioredoxin reductase 1) inhibitor, enhanced the sensitivity of PCK1-deficient HCC cells to sorafenib-induced apoptosis [74]. Targeting PCK1 and paclitaxel synergistically inhibited the growth of triple-negative breast cancer (TNBC) [52]. The PCK1-specific inhibitor 3-MPA combined with vilofinil significantly enhanced drug sensitivity [106].

**Fig. 3:**
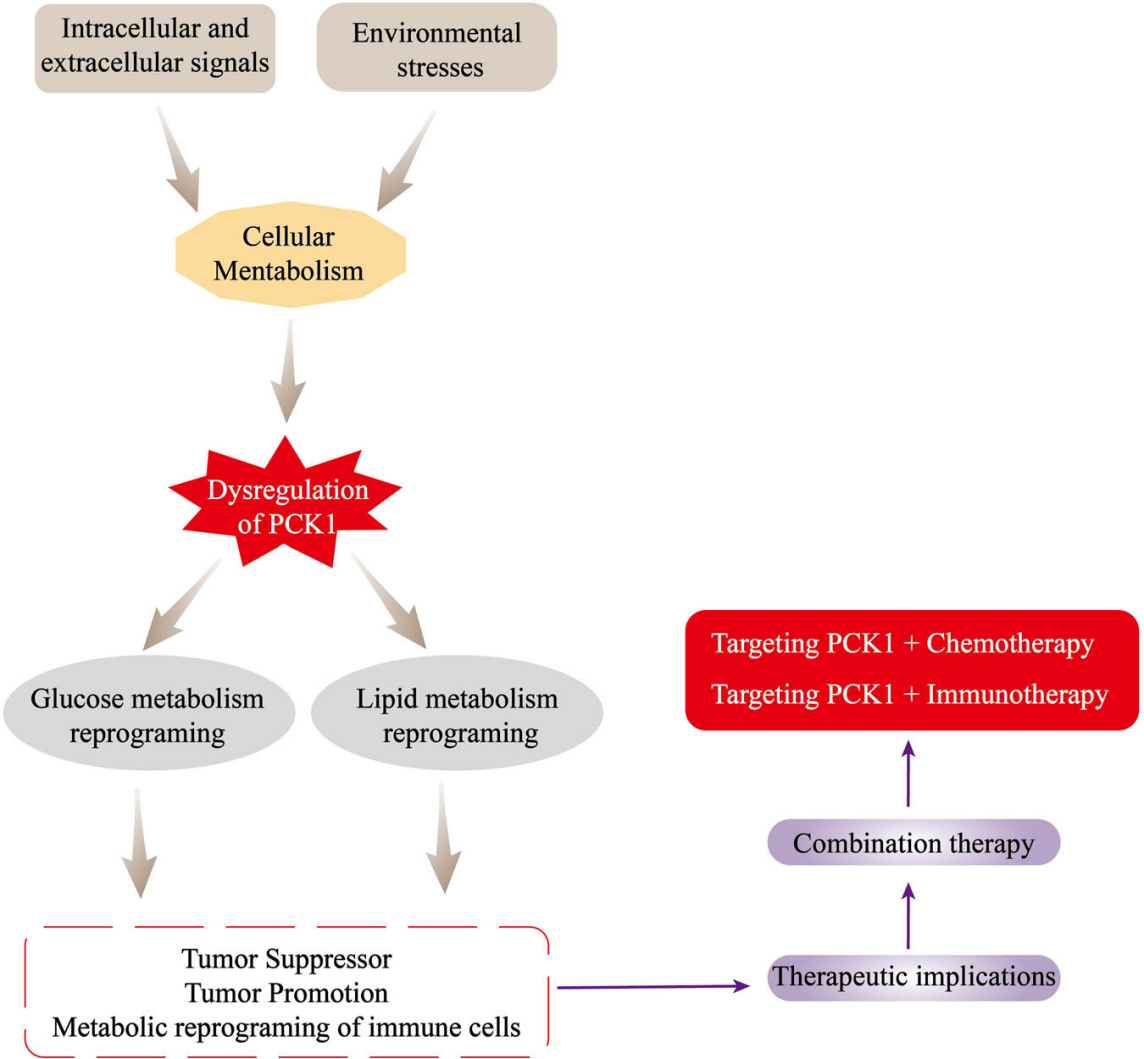
PCK1-Targeted combination therapy for cancer treatment. Intracellular and extracellular signaling events and environmental stresses can affect PCK1 activity by regulating cell metabolism, leading to metabolic reprogramming, and thus affecting the occurrence and development of cancer. Targeting PCK1 combined with chemotherapy and immunotherapy are potential treatment strategies.

Tumor immunotherapy is becoming a treatment method that may lead to important innovations in the field of cancer treatment [115]. Multiple types of immune cells, especially T cells, are involved in tumor immune surveillance [115]. Therefore, adoptive cell therapy with tumor-specific T cells (ACT) has emerged as a promising form of anticancer immunotherapy for increasing the number of cytotoxic T cells to suppress tumor growth [115, 116]. ACT is the core technology of tumor immunotherapy and involves the expansion of T cells in vitro. However, due to repeated stimulation and activation, a large number of expanded T cells gradually enter a state of exhaustion and eventually die after infusion, which makes it difficult to achieve the ideal therapeutic effect [117–119]. Therefore, T cells need to be guaranteed a long cell life span to cope with the demands of reawakening with antigens. There is metabolic competition between T cells and tumor cells in the TME [119]. Metabolic reprogramming of T cells by PCK1 can repair and increase the anti-tumor immune function of T cells and limit tumor growth [21, 30]. We investigated whether combining PCK1 with immune checkpoint inhibition will solve the problems of immune tolerance and poor sensitivity. Future research should focus on this aspect, which may reveal a potential new method of cancer immunotherapy. These studies provide new directions for clinically targeted combination therapy in cancer patients. At present, many drugs targeting PCK1 for cancer treatment remain in the preclinical stage and have not entered clinical trials. Overall, targeted metabolic therapy may also be a promising strategy for cancer treatment in combination with chemotherapy and immunotherapy in the future (Fig. 3).

## Discussion and perspective

The diversity of PCK1 expression in different cancer models makes it a challenging therapeutic target. Therefore, the underlying molecular mechanisms of PCK1 development in different tumor tissues and disease stages should be fully investigated. Overall, we discussed that PCK1 is downregulated in tumors of gluconeogenic organs (HCC and RCC) and has anti-tumor effects. In contrast, PCK1 is upregulated and oncogenic in tumors originating from non-gluconeogenic organs (CRC, lung cancer, melanoma, breast cancer, pancreatic cancer, and gastric cancer). The different tissues of tumor origin and the autonomous energy demand of tumors for proliferation and metastasis lead to metabolic heterogeneity, which may be one of the key reasons why PCK1 has different functions in different organs. Regulating PCK1 expression and activity in various cancers involves interplay between metabolic and oncogenic pathways. PCK1 and its related metabolic pathways regulate a variety of cellular functions, including biosynthetic pathways and immune responses, as well as inducing metabolic reprogramming to produce macromolecules and energy, maintaining redox homeostasis, and activating signal transduction. Hence, these may represent interesting targets for cancer therapy. In the future, scholars should continue to explore the unknown functions of PCK1 beyond metabolism to provide fundamental insights and novel therapeutic strategies for human malignancies.

At present, probing the unique metabolic changes in cancer remains a great challenge. Considering the expression, activity, and functional differences of PCK1 in various tumor types, precision, and personalization are important development directions for metabolic therapy. Establishing targeted treatment strategies for different metabolic types of tumors, dynamically adjusting them according to the metabolic adaptation of tumors, and combining effective treatment strategies may be the future direction of tumor metabolic therapy. However, in addition to metabolic heterogeneity, the challenges of targeting metabolism to treat cancer include the following. (1) The plasticity of tumor metabolism will also become a major challenge: tumor cells may compensate for certain metabolic limitations through metabolic reprogramming, which will affect the efficacy of metabolic therapy. It is necessary to track changes in tumor metabolism over time and switch treatment strategies. (2) Tumor cells reconstitute metabolic pathways, leading to the development of drug resistance and reducing the effectiveness of precision-targeted therapy. Consequently, combination therapy or treatment that blocks multiple pathways has advantages over monotherapy. (3) The side effects are obvious; most metabolic pathways in tumor cells and normal cells are common, and targeting tumor cells interferes with normal cells and may lead to significant toxic effects. Additionally, metabolic therapies targeting tumors may have an impact on cells in the TME, impair immune cell function, and inhibit anti-tumor immunity. Currently, a series of preclinical and clinical studies targeting metabolic enzymes for cancer therapy are ongoing and require comprehensive and careful monitoring and evaluation. Accelerating the progress of tumor metabolic therapy and exploring better therapeutic strategies targeting tumor metabolism are urgently needed.
